# Prenatal tobacco smoke exposure predisposes offspring mice to exacerbated allergic airway inflammation associated with altered innate effector function

**DOI:** 10.1186/s12989-017-0212-6

**Published:** 2017-08-22

**Authors:** Maria Ferrini, Sophia Carvalho, Yoon Hee Cho, Britten Postma, Lucas Miranda Marques, Kent Pinkerton, Kevan Roberts, Zeina Jaffar

**Affiliations:** 1 Center for Environmental Health Sciences, Biomedical and Pharmaceutical Sciences, College of Health Professions and Biomedical Sciences, University of Montana, Missoula, MT MT 59812 USA; 2 Department of Anatomy, Physiology and Cell Biology, Center for Health and the Environment, University of California, Davis, CA USA

**Keywords:** Prenatal exposure, Environmental tobacco smoke, Allergic asthma, Innate immunity

## Abstract

**Background:**

Epidemiological studies suggest that prenatal and early life environmental exposures have adverse effects on pulmonary function and are important contributors in the development of childhood asthma and allergic disease. The mechanism by which environmental tobacco smoke (ETS) exposure in utero promotes the development of allergic asthma remains unclear. In this study, we investigated the immunological consequences of prenatal exposure to ETS in order to understand events responsible for the development or exacerbation of allergic asthma.

**Methods:**

Pregnant C57BL/6 mice were exposed to either ETS or filtered air throughout gestation and the effect on pulmonary inflammation in the offspring were examined and compared. Specifically, the effects on eosinophilic inflammation, airway hyperreactivity, goblet cell hyperplasia, properties of pulmonary natural killer (NK) cells and type 2 cytokines elicited in response to inhaled house dust mite (HDM) allergen were investigated in the progeny.

**Results:**

Exposure to ETS prenatally significantly exacerbated HDM-induced airway eosinophilic inflammation, hyperreactivity, mucus secretion, cysteinyl leukotriene biosynthesis and type 2 cytokine production in the offspring. Consistently, lung mononuclear cells from ETS-exposed offspring secreted higher levels of IL-13 when stimulated in vitro with anti-αβ TCR antibody or HDM allergen. Moreover, offspring from ETS-exposed dams exhibited a higher frequency of CD11b^+^ dendritic cells and CD3^+^CD4^+^ T lymphocytes in the lungs following allergen inhalation compared to air-exposed mice. Unexpectedly, the exacerbated allergic inflammation in the ETS-exposed offspring was associated with a reduction in CD3^−^CD19^−^NK1.1^+^CD94^+^ NK cell numbers and their IFN-γ production, highlighting a role for altered innate immunity in the enhanced allergic response.

**Conclusion:**

Our results reveal that prenatal exposure to ETS predisposes offspring to an exacerbated allergic airway inflammation that is associated with a reduction in pulmonary NK cell function, suggesting that NK cells play a key role in controlling asthma severity.

## Background

Allergic asthma is the most common form of asthma in children [1] characterized by bronchial eosinophilic inflammation, airway hyperreactivity (AHR), type 2 cytokine production, IgE synthesis, mucus hypersecretion and remodeling [2, 3]. The prevalence of asthma has increased in the past few decades and currently affects one in ten children [4]. While genetic factors contribute to susceptibility and development of asthma, the increased disease prevalence cannot be completely explained on the basis of genetics. Accumulating epidemiological evidence suggests that environmental factors, such as air pollution and exposure to secondhand or environmental tobacco smoke (ETS), are key contributors in the development of childhood asthma [5–7]. Specifically, maternal smoking is strongly linked to allergic asthma and respiratory infection in children [8–11].

Cigarette smoking has a major impact on respiratory health and is a key risk factor for pulmonary dysfunction in both adults and children. Smoke exposure during fetal development and in the early years of a child’s life is strongly associated with respiratory tract infections and asthma that can persist into adulthood [9]. Exposure to cigarette smoke is known to impact the immune system [12], but how this predisposes children or adults to developing chronic inflammatory diseases is unclear. Despite continuing efforts to reduce smoking prevalence, over one billion people worldwide remain smokers, with globally half of all children estimated to be exposed to secondhand smoke [13].

The effect of maternal smoke exposure in promoting the development of asthma and the mechanism responsible for this process remains elusive. Our previous work demonstrated that in utero ETS exposure of mice alters DNA methylation patterns and increases airway hyperreactivity in the offspring [14]. In this study, we examined whether prenatal ETS exposure was associated with increased risk of development or exacerbation of asthma using a model of house dust mite (HDM) allergen inhalation [15, 16]. We have recently shown that pulmonary NK cells play a key role in limiting allergic inflammatory responses to inhaled HDM allergen [15, 16]. Thus, we specifically investigated the impact of gestational ETS exposure on pulmonary innate and inflammatory responses in the offspring. Our data revealed that prenatal exposure to ETS caused an exacerbated pulmonary eosinophilic inflammation, AHR, airway mucus secretion and raised serum immunoglobulin (Ig)E levels in the progeny following allergen inhalation. In addition, there was a pronounced increase in allergen-induced production of cysteinyl leukotrienes (cysLT) and type 2 cytokines including interleukin (IL)-13 in the airways. The exacerbated inflammatory response was accompanied with a marked reduction in the number of natural killer (NK) cells in the lung tissue and an impaired interferon (IFN)-γ production by the pulmonary NK cells. Collectively, these results demonstrate that prenatal ETS exposure promotes an exacerbated allergic airway inflammation in the offspring associated with a reduction in pulmonary NK cells and IFN-γ production but a heightened ability of the lungs to mount a Th2 response. This study provides important insight into how prenatal ETS exposure influences susceptibility to allergic asthma and suggests that NK cells play a key role in modulating this process.

## Methods

### Animals

C57BL/6 mice were purchased from Harlan Laboratories (Indianapolis, IN) and maintained in pathogen-free conditions in a barrier facility at either the University of California–Davis (Davis, CA) or the University of Montana (Missoula, MT). Mice were treated in accordance with NIH guidelines and the American Association of Laboratory Animal Care regulations and all animal experiments approved by University of Montana Institutional Animal Care and Use Committee.

### ETS exposure

**Fig. 1:**
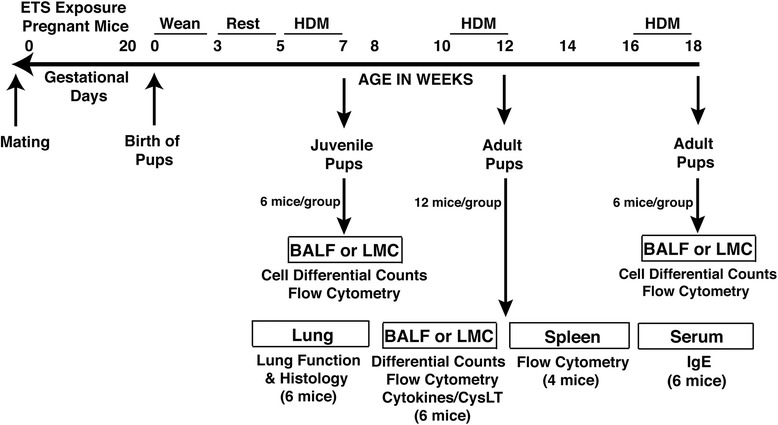
The experimental design of the study, ETS exposure and timeline of HDM challenges. Timed-pregnant C57BL/6 female mice were exposed to either ETS (daily to 1.0 mg/m^3^ for 6 h/day) or filtered air throughout the gestation period. Once the dams gave birth, both dams and pups were only exposed to filtered air until weaning. The offspring mice (at 5, 10 and 16 weeks old) were then acutely challenged intranasally with HDM allergen or PBS (6–12 mice per treatment group) over a period of two weeks before being studied at 7, 12 and 18 weeks of age as illustrated and all experiments were performed at least twice

The study consisted of mating 2 female C57BL/6 mice by pairing with 1 male (2 Female: 1 Male) per cage to create a timed-pregnant exposure set-up. Following confirmation of a vaginal plug, pregnant female mice were exposed to either environmental tobacco smoke (ETS) or filtered air throughout gestation. ETS was generated by a smoke exposure system (Dr. Kent Pinkerton Laboratory, University of California-Davis). For the ETS exposed group, timed-pregnant mice were exposed daily to an approximate concentration of 1.0 mg/m^3^ of tobacco smoke for 6 h/day using 3R4F research cigarettes (Tobacco Research Institute, University of Kentucky, Lexington, KY) that were burned at a rate of two cigarettes every 10 min with a puff volume of 35 mL over a duration of 2 s, once per minute. Both side-stream and mainstream cigarette smoke were collected via a chimney and passed to a dilution and aging chamber to achieve the target concentration of ETS. After each exposure to ETS for 6 h, the pregnant mice were then kept in filtered air. For the control group, timed-pregnant mice were handled in the same way but exposed to filtered air for 24 h 7 d/week for the duration of the study. The concentration of carbon monoxide in the exposure chambers was monitored and kept to 4.8 ± 0.8 ppm. It is important to note that a person actively smoking can attain particulate levels as high as 2.0 mg/m^3^ [17]. Therefore, in order to sustain concentrations relevant to human exposure levels, the total concentration of suspended particulates was maintained at 1.0 ± 0.17 mg/m^3^ for this study. Once the dams gave birth, the dams and pups were only exposed to filtered air until weaning and then transported to the University of Montana by plane. The offspring were given 2 weeks rest to adjust to their new environment before being studied. Figure 1 illustrates the experimental design for this study. The number of pups in a litter for each group was recorded. ETS exposure did not induce any spontaneous losses in mice. The litter size (6.7 vs. 7.1, mean for air-exposed and ETS-exposed dams, respectively) and sex-ratio (12.7:13.3 vs. 14.8:13.2, male:female for air- and ETS-exposed, respectively) were not significantly different between the two groups.

### HDM allergen challenge

Offspring mice (adult and juvenile pups) were challenged with HDM allergen over a period of two weeks using an acute model of allergic airway inflammation previously described by our laboratory and other investigators [15, 16, 18]. Mice were lightly anesthetized with isofluorane to allow intranasal instillation of 30 μl solution of HDM allergen extract (Dermatophagoides pteronyssinus, Greer Laboratories) in sterile PBS, or administration of PBS alone (control) over a period of 2 weeks. Briefly, mice were first sensitized with HDM (100 μg) by intranasal instillation on Day 0 and then challenged with the allergen (50 μg) on Days 7 and 14. Forty-eight hours after the last exposure (Day 16), pulmonary function and the level of airway inflammation were determined.

### Lung function

Lung respiratory resistance (R_L_, cm H_2_O.s/ml) and dynamic compliance (C_Dyn_, ml/cm H_2_O) was measured in anesthetized and tracheotomized mice that were mechanically ventilated in response to increasing concentration of methacholine inhalation (1.5–24 mg/ml) using the pulmonary function equipment by Buxco Research Systems.

### Level of pulmonary inflammation

Bronchoalveolar lavage was performed (using 3 × 0.5 ml PBS) to collect bronchoalveolar lavage fluid (BALF) for analysis. BALF was cooled on ice and centrifuged at 4C for 10 min. The cell pellets were resuspended in PBS and the supernatants frozen (−80C) for analysis of cysteinyl leukotriene and cytokine production. Eosinophil peroxidase (EPO) levels in the bronchoalveolar lavage cells were determined by colorimetric analysis as we have previously described [19]. Cells were centrifuged (Cytospin II, Shandon) onto glass slides and stained with hematoxylin and eosin (Hema 3, Fisher Scientific, Waltham, MA, USA). Differential counts of airway inflammatory cells (alveolar macrophages, lymphocytes, eosinophils and neutrophils) were performed by light microscopic evaluation of Hema3-stained cytospin preparations. At least 350 cells were counted in 5 different fields of view (based on standard morphological criteria) and results expressed as absolute cell number (calculated by multiplying the total leukocyte number by the percentage of each population of interest). Lung tissue was dispersed by collagenase (Type IV; Sigma-Aldrich), and lung mononuclear cells (LMC) were isolated by Percoll (Sigma-Aldrich) density gradient for functional analysis.

### Histological determination of peribronchial inflammation and goblet cell hyperplasia

Lung tissue was fixed in 4% paraformaldehyde and embedded in paraffin using a Leica ASP 300 tissue processor (Leica, Bannockburn, IL). Microtome sections were cut at 5-μm thickness and stained with hematoxylin and eosin (H&E) using a Shandon Varistain 24–4 (Thermo Fisher Scientific). Alternatively, sections were stained using periodic acid-Schiff (PAS) reagent. The level of peribronchial inflammation (H&E stained) or mucus production (PAS stained) was analyzed by microscopy and the transmitted light images were collected on a Nikon Eclipse 800 microscope equipped with an Olympus DP 71 camera and cellSens software (Version 1.9).

### Flow cytometry

To examine inflammatory cells in the lungs and spleen, BALF, LMC or splenic cells were FcγR blocked using 2.4G2 antibody (ATCC) and stained with combinations of the following mouse conjugated mAb (all from BioLegend, San Diego, CA) using standard single step staining protocol: allophycocyanin (APC) or fluorescein isothiocyanate (FITC) anti-CD3 and APC/Cy7 anti-CD4 (to stain CD3 + CD4+ T cells); APC-Cy7 anti-CD19, PE anti-CD94 (NKG2), APC or phycoerythrin (PE) anti-CD49b DX5 (pan-NK cells) and FITC or APC anti-NK1.1 (PK136) (to stain NK cells); PE, FITC or Brilliant Violet 421 anti-CD11b, PE or APC/Cy7 anti-I-A/I-E and APC or PE anti-CD11c (to stain dendritic cells), APC/Cy7 anti-Ly-6G/Ly6C (Gr-1), APC or PE anti-F4/80, and PE anti-Siglec-F (BD Biosciences, to stain eosinophils). Flow cytometric acquisition was performed on a FACSAria II (BD Biosciences) by 5-color analysis using FACSDiVa software and FlowJo, with a minimum of 50,000 live, single-cell events per sample collected.

### Measurement of cytokines, cysteinyl leukotrienes (cysLT) and serum IgE

BALF cytokine levels were determined using ELISA (for measurement of IL-13) or sensitive V-Plex Pro-Inflammatory Panel-1 assay (for measurement of IL-4, IL-5 and IL-6, MesoScale Discovery). In addition, BALF cysLT (Cayman Chemical Company, Ann Arbor, MI) and serum IgE (BioLegend) levels were measured using ELISA, according to manufacturer’s instructions. To examine cytokine production in vitro, LMC (1 × 10^6^ cells/ml prepared by enzymatic dispersion of lung tissue were stimulated with immobilized anti-αβ TCR (2 μg/ml, H57, ATCC), HDM allergen (20 μg, Greer Laboratories) or media alone. After culture for 24 h, supernatants were harvested and IL-13 production measured by ELISA (R&D Systems). In addition, LMC NK cells were stimulated with anti-NK1.1 antibody (PK136, 20 μg/ml) or media alone, in the presence of 10 ng/ml IL-2 (R&D Systems). After culture for 24 h, supernatants were harvested and IFN-γ production measured by ELISA (R&D Systems).

### Statistical analyses

Data were analyzed using GraphPad Prism 5.0 (GraphPad, La Jolla, CA). Results involving two variables were analyzed by two-way ANOVA with a Bonferroni post-test. Data comparing two groups were analyzed using an unpaired t test. Data shown are mean ± SEM. A *p* value <0.05 was considered statistically significant.

## Results

### Prenatal ETS exposure promoted a protracted predisposition to exacerbated allergic airway inflammation in offspring mice

**Fig. 2:**
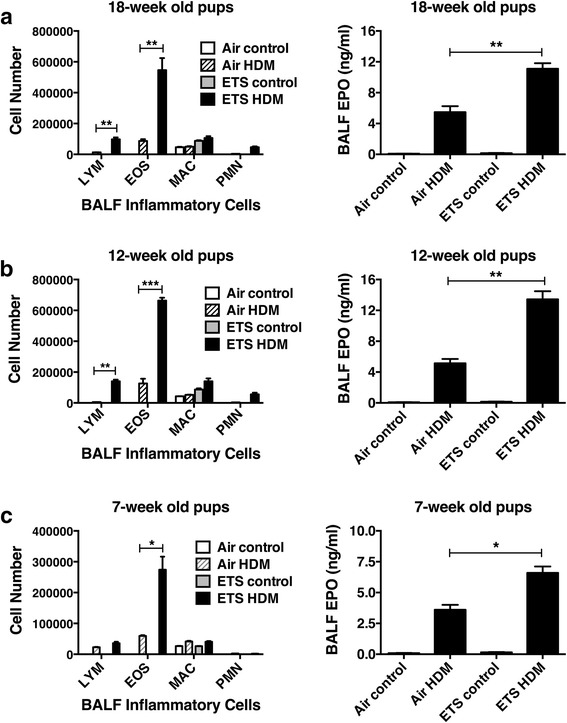
Prenatal ETS exposure promotes a protracted predisposition to exacerbated allergic airway inflammation in the progeny. The effect of exposure to prenatal ETS or filtered air on the exacerbation of allergic airway inflammation was examined in **a** 18-week old, **b** 12-week old and **c** 7-week old C57BL/6 pups. The offspring mice (6 per group) were intranasally challenged with HDM allergen or PBS (control) and bronchoalveolar lavage fluid (BALF) was collected for analysis. Cell differential counts were determined and expressed as absolute cell numbers per mouse of lymphocytes (LYM), macrophages (MAC), eosinophils (EOS), and polymorphonuclear neutrophils (PMN). Eosinophil peroxidase (EPO) levels were assessed by colorimetric analysis. Results are mean ± SEM (*n* = 6) and representative of at least two independent experiments, ***p < 0.001, **p < 0.01 and *p < 0.05

Pregnant C57BL/6 female mice were exposed to either ETS or filtered air (4 female mice per group) throughout gestation. ETS was generated by a tobacco smoke exposure system and pregnant mice were exposed daily to 1.0 mg/m^3^ of ETS for 6 h/day. The experimental design, ETS exposure and timeline of HDM challenges are illustrated in Fig. 1 that highlights evaluation of pups at 7, 12 and 18 weeks of age. The adverse effects of prenatal exposure to ETS or filtered air on pulmonary inflammation was assessed in both adult and juvenile offspring mice after an acute sensitization and challenge with intranasal HDM allergen over a period of two weeks using a model of allergic asthma that we have previously developed [15]. Control mice were not challenged with HDM allergen but treated with PBS instead. Prenatal ETS exposure caused a pronounced elevation in the number of eosinophils, lymphocytes and level of cell-associated eosinophil peroxidase (EPO) in the airways of both 18- and 12-week old offspring after allergen inhalation (Fig. 2a, b). However, the number of polymorphonuclear neutrophils (PMN) and macrophages did not significantly differ between the ETS- and air-exposed mice. Similarly, an exacerbated eosinophilia was also observed in the airways of juvenile 7-week old pups prenatally exposed to ETS (Fig. 2c), although fewer numbers of inflammatory cells were detected in the BALF compared to the adult mice, likely reflecting the smaller size of these young mice. Notably, in the absence of HDM inhalation (control mice), the level of inflammatory cells in the airways of ETS- and air-exposed pups was low (Fig. 2). Collectively, these results show that in utero ETS exposure not only predisposes offspring to exacerbated allergic pulmonary inflammation but also promotes a protracted predisposition (at least up to 18 weeks) to allergic airway disease.

**Fig. 3:**
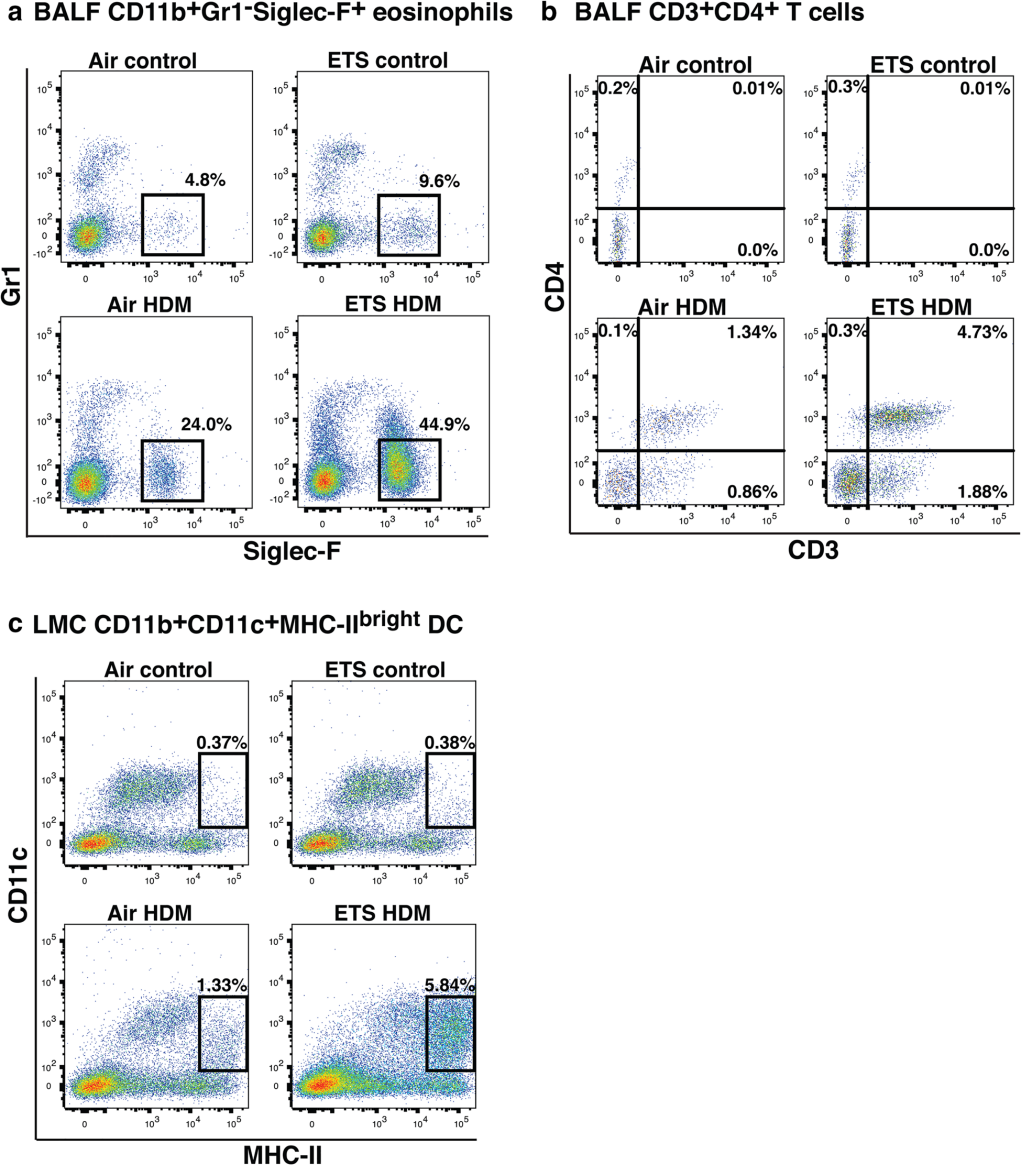
Prenatal ETS exposure exacerbates allergen-induced accumulation of inflammatory cells in the lungs of the offspring. The effect of prenatal ETS or filtered air exposure on Siglec-F^+^ eosinophilis, CD4^+^ T cells and monocyte-derived dendritic cells (DC) accumulation in the lungs was investigated in 12-week old offspring. Mice (6 per group) were intranasally challenged with HDM allergen or PBS (control). Bronchoalveolar lavage fluid (BALF) was collected, and lung mononuclear cell (LMC) isolated by collagenase dispersion of lung tissue for analysis. The frequency of **a** CD11b^+^Gr1^−^Siglec-F^+^ eosinophils (after gating on CD11b^+^ and F4/80^−^ cells), **b** CD3^+^CD4^+^ T cells in the BALF, and **c** CD11b^+^CD11c^+^MHC-II^bright^ dendritic cells (DC) in the LMC was determined using multicolor flow cytometry. Data are representative of at least two independent experiments

To more fully characterize the exacerbated pulmonary inflammatory response, our subsequent analysis focused on dissecting the allergic response in the 12-week old pups only. Consistent with the BALF cell differential counts, flow cytometric analysis of BALF cells revealed a pronounced increase in the number of BALF CD11b^+^Siglec-F^+^ eosinophils after HDM inhalation in the prenatal ETS-exposed mice compared to air-exposed controls (44.8% in ETS-exposed vs 24.0% in air-exposed pups, Fig. 3a). Remarkably, in utero ETS exposure alone (i.e. baseline levels in the absence of allergen challenge) caused a mild increase in Siglec-F^+^ eosinophils (9.6% in ETS-exposed vs 4.8% in air-exposed). We further examined the effect of prenatal ETS exposure on the frequency of T cells and monocyte-derived dendritic cells (DC) in the lungs. Pulmonary DC are crucially involved in allergen sensitization and play an important role in the development of Th2-mediated allergic airway inflammation [20]. Our data revealed that the frequency of CD3^+^CD4^+^ T cells in the airways was also increased in ETS-exposed compared to air-exposed offspring after HDM inhalation (4.73% in ETS-exposed vs 1.34% in air-exposed, Fig. 3b). Similarly, an elevation in the frequency of pulmonary CD11b^+^CD11c^+^MHC-II^bright^ DC was observed in ETS-exposed group following allergen challenge (5.84% in ETS-exposed vs 1.33% in air-exposed, Fig. 3c). It is important to note that, in the absence of HDM challenge (control mice), the frequency of CD4^+^ T cells and CD11b^+^ DC in the lungs of both ETS- and air-exposed offspring was low.

### Prenatal ETS exposure caused an exacerbated allergen-induced peribronchial inflammation, mucus secretion, AHR and serum IgE production

**Fig. 4:**
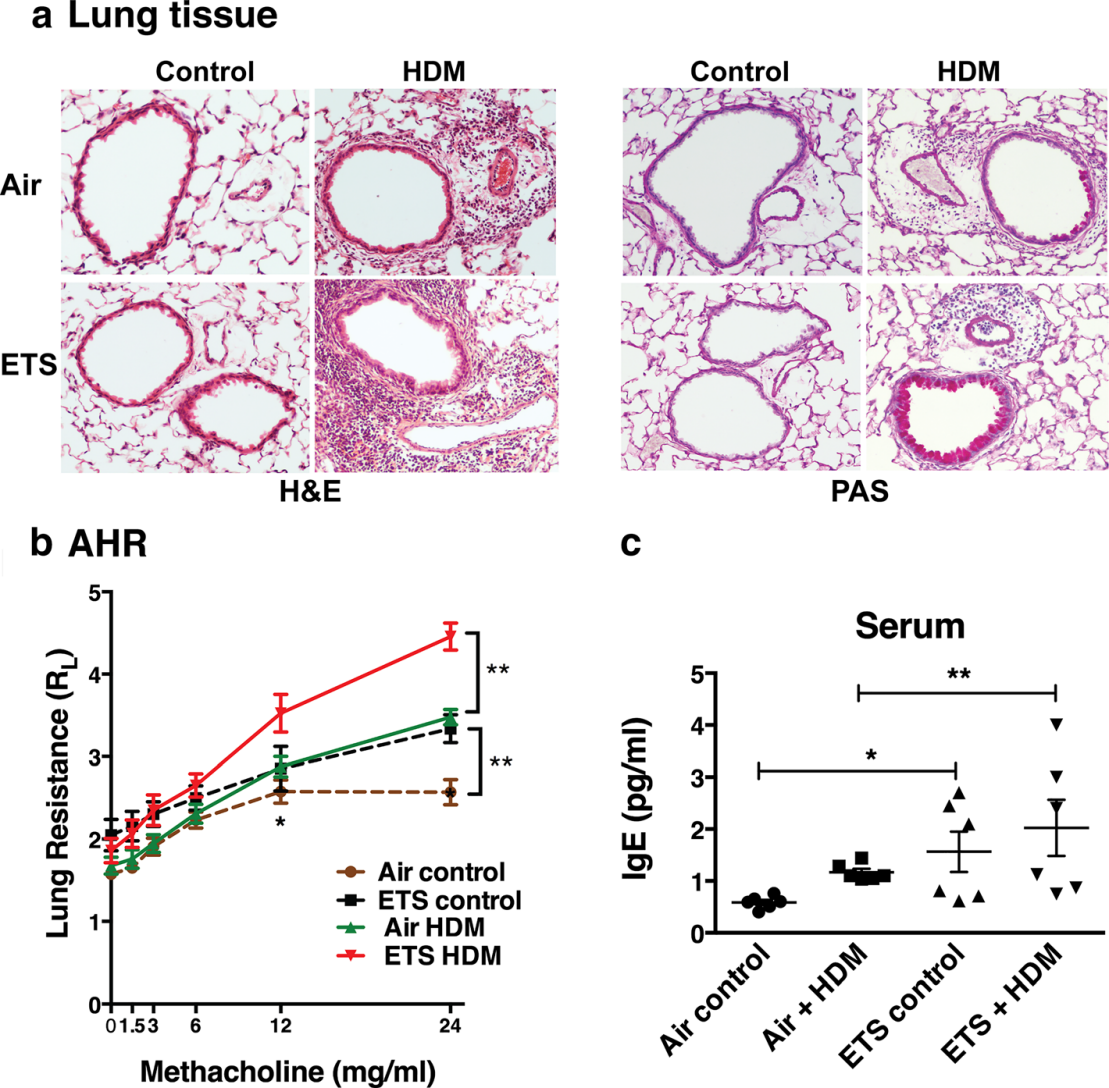
Prenatal ETS exposure exacerbates allergen-induced peribronchial inflammation, mucus secretion and AHR in the offspring. The effect of exposure to prenatal ETS or filtered air on the exacerbation of the cardinal features of asthma was examined in 12-week old pups. Offspring (6 mice per group) were intranasally challenged with HDM allergen or PBS (control) and lung tissue and serum collected for analysis. **a** Peribronchial inflammation and goblet cell hyperplasia were determined by histological analysis of H&E-stained and PAS-stained lung segments, respectively (20×). **b** AHR was assessed by measurement of lung resistance using Buxco System. **c** Serum IgE levels were measured by ELISA. Results are mean ± SEM (*n* = 6), **p < 0.01 and *p < 0.05. Data are representative of at least two independent experiments

We next assessed the adverse effects of prenatal ETS exposure on a number of prominent features of asthma, namely peribronchial inflammation, goblet cell hyperplasia, AHR and IgE production. Histological analysis of lung tissue sections stained with H&E or PAS revealed a marked increase in allergen-induced peribronchial inflammation and mucus production in prenatal ETS-exposed 12-week old offspring compared to the air-exposed controls (Fig. 4a, b). In the absence of allergen challenge (baseline control), negligible peribronchial inflammation or mucus production was observed in the lung tissue of either ETS or air-exposed offspring. In addition to the exacerbated inflammatory response, an elevated AHR and serum IgE levels were also observed in the prenatal ETS-exposed pups when compared to air-exposed offspring after HDM inhalation (Fig. 4c, d). Interestingly, prenatal ETS exposure alone (i.e. ETS control in the absence of allergen challenge) caused a mild increase in both AHR and serum IgE levels when compared to air-exposed controls (Fig. 4c, d). Collectively, these results reveal that in utero ETS exposure predisposes offspring to exacerbated allergic airway disease characterized by enhanced airway inflammation, goblet cell hyperplasia, AHR and raised IgE levels.

### Prenatal ETS exposure resulted in a heightened type 2 cytokine and cysteinyl leukotriene production in the lungs of offspring after allergen inhalation

**Fig. 5:**
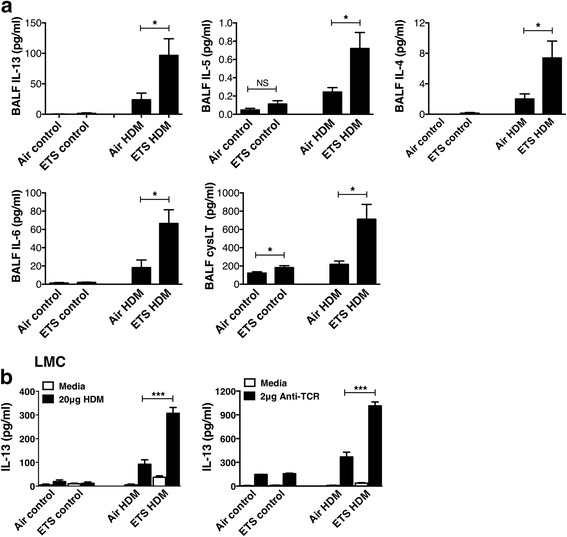
Prenatal ETS exacerbates allergen-induced production of type 2 cytokines and cysteinyl leukotrienes in the offspring. The effect of exposure to prenatal ETS or filtered air on the exacerbation of type 2 cytokine and cysLT production in the airways was examined in 12-week old pups. The offspring mice (6 per group) were intranasally challenged with HDM allergen or PBS (control) and bronchoalveolar lavage fluid (BALF) and lung tissue collected for analysis. Lung mononuclear cells (LMC) were isolated by collagenase dispersion of lung tissue. **a** Cytokine levels in the BALF were measured using ELISA or V-Plex assay and cysteinyl leukotriene (cysLT) levels determined using ELISA. **b** LMC stimulated with either HDM allergen extract (20 μg, Greer Laboratories) or immobilized anti-αβ TCR antibody (2 μg/ml, H57, ATCC). After culture for 24 h, production of IL-13 was measured in the supernatants using ELISA. Results are mean ± SEM (*n* = 6), *p < 0.05 and ***p < 0.001. NS = not significant

Asthma is characterized by type 2 immune responses and production of cytokines such as IL-4, IL-5 and IL-13 that drive the allergic disease. To examine events responsible for exacerbated allergic response following prenatal ETS exposure, we assessed whether the exposure promotes generation of type 2 cytokine or cysteinyl leukotriene (cysLT) in the airways. Our data revealed that prenatal ETS exposure caused markedly elevated HDM-induced levels of IL-13, as well as IL-4, IL-5 and IL-6 in the BALF of 12-week old pups. These levels were significantly higher (approximately 3-fold) than that observed in filtered air-exposed offspring after allergen inhalation (Fig. 5a). Little or no production of type 2 cytokines was observed in the airways of both ETS-exposed and air-exposed control mice that did not inhale HDM allergen (controls). The cysLT, LTC_4_, LTD_4_, and LTE_4_, are peptide-conjugated lipids generated from arachidonic acid by the action of enzyme 5-lipoxygenase [21] which are produced by various inflammatory cells, notably macrophages, mast cells, eosinophils and activated DC [22]. Originally identified on the basis of their contractile properties on intestinal and bronchial smooth muscle [23], they are now recognized as potent inflammatory mediators that initiate and propagate a diverse array of biologic responses and play an essential role in allergic responses to HDM allergen [22]. We therefore investigated the effect of prenatal ET exposure on cysLT levels in the BALF of offspring mice after allergen inhalation. Consistent with the exacerbated Th2 cytokine production, the level of allergen-induced cysLT was significantly elevated following HDM challenge in the airways of ETS-exposed offspring compared to filtered air group (856.9 ± 199.2 pg/ml in ETS-exposed vs 184.7 ± 34.4 pg/ml in air-exposed, Fig. 5a). A slight increase in cysLT was also observed in the airways of ETS-exposed controls when compared to filtered air mice in the absence of allergen challenge (PBS controls). In summary, these results demonstrate that prenatal ETS exposure predisposes offspring to exacerbated type 2 cytokine and cysLT production in the airways. In agreement with BALF cytokines, LMC from allergen challenged ETS-exposed pups exhibited highly exacerbated IL-13 production when stimulated in vitro with either HDM extract or anti-αβ TCR antibody (Fig. 5b). These results reveal that in utero ETS exposure skews immune responses to promote Th2 inflammation in the lungs of the progeny.

### Prenatal ETS exposure caused a striking reduction in the frequency of pulmonary NK cells and their production of IFN-γ

**Fig. 6:**
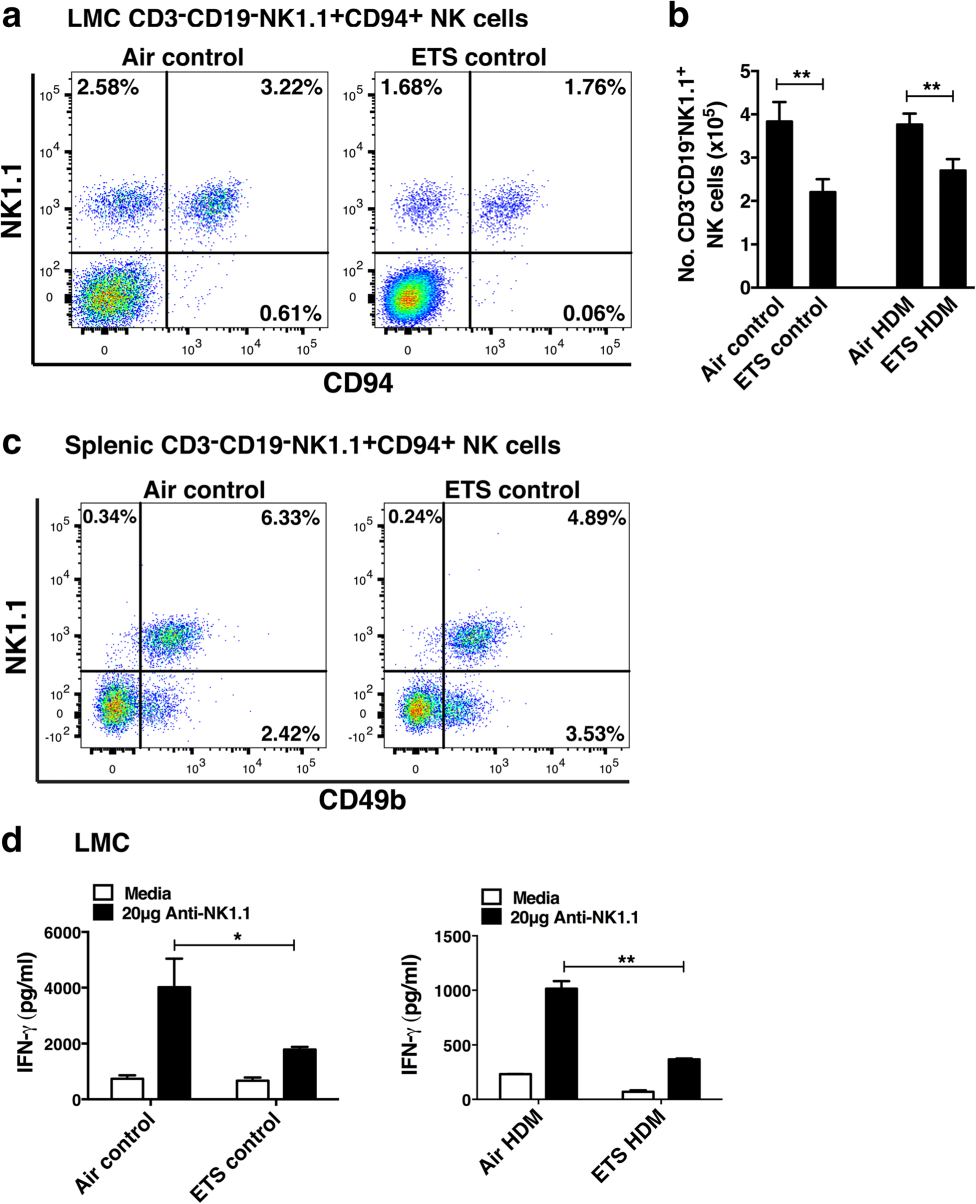
Prenatal ETS exposure causes a reduction in pulmonary NK cell numbers and their IFN-γ production. The effect of exposure to prenatal ETS or filtered air on lung and splenic natural killer (NK) cell numbers and IFN-γ was examined in 12-week old pups. Offspring (6 mice per group) were intranasally challenged with HDM allergen or PBS (control). Lung mononuclear cells (LMC) were isolated by collagenase dispersion of lung tissue. **a** After gating on CD3^−^CD19^−^ cells, the frequency of LMC CD3^−^CD19^−^NK1.1^+^CD94^+^ NK cells was determined using multicolor flow cytometry and **b** expressed as total number of NK cells (per mouse). **c** Frequency of splenic CD3^−^CD19^−^NK1.1^+^CD94^+^ NK cells was determined using multicolor flow cytometry. **d** NK cells in LMC were stimulated with anti-NK1.1 antibody (PK136, 20 μg/ml) for 24 h and IFN-γ production measured in the supernatants by ELISA. Results are mean ± SEM (*n* = 4), *p < 0.05 and **p < 0.01. Data are representative of at least 2 independent experiments

**Fig. 7:**
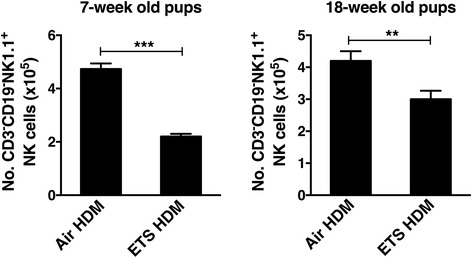
Prenatal ETS causes a reduction in pulmonary NK cell numbers in juvenile and adult mice. The effect of exposure to prenatal ETS or filtered air on pulmonary natural killer (NK) cell numbers was examined in both juvenile (7-week old) and adult (18-week old) offspring mice following intranasal challenge with either HDM allergen or PBS (control). After gating on CD3^−^CD19^−^ cells, the number of CD3^−^CD19^−^NK1.1^+^ NK cells present in the lung mononuclear cells was determined using multicolor flow cytometry and expressed as total number of NK cells (per mouse). Results are mean ± SEM (*n* = 4), **p < 0.01, ***p < 0.001

Although initially described as lymphocytes involved in innate immunity, NK cells are now known to be involved in the regulation of adaptive immune responses [24]. Indeed, NK cells have long been known to play critical roles in host defense against pathogens and tumors through their cytotoxic activity and cytokine production [24–26]. In this study, the effect of prenatal ETS exposure on tissue and peripheral NK cells was assessed using NK1.1 alloantigen (a member of the Mkrp1c gene family) as a specific marker for NK cells [27], and CD94/NKG2 which is a family of C-type lectin receptors predominantly expressed on the surface of NK cells. NK cells in the lungs of prenatal ETS-exposed offspring were enumerated and compared with air-exposed group using multi-color flow cytometric analysis (after exclusion of CD3^+^ T cells and CD19^+^ B cells). Unexpectedly, approximately two-fold reduction in the frequency of CD3^−^CD19^−^NK1.1^+^CD94^+^ NK cells was found in the lungs of offspring prenatally exposed to ETS compared to filtered air group (1.76% in ETS-exposed vs 3.22% in air-exposed) (Fig. 6a and b) as well as in the spleen (Fig. 6c). The reduced total number of pulmonary NK cells was still evident following the onset of HDM-induced inflammation (Fig. 6b) and was a stable defect observable at all ages examined (Fig. 7). Although largely recognized by their ability to mediate cytolytic activity, NK cells are also an important source of pro-inflammatory cytokines such as IFN-γ. To examine effect of prenatal ETS exposure on cytokine production by NK cells, LMC were stimulated with anti-NK1.1 antibody. The data revealed that LMC from control offspring prenatally exposed to ETS (that were not challenged with HDM) produced significantly less IFN-γ (2-fold reduction) compared to air-exposed group (1785.6 pg/ml in ETS-exposed vs 4015.3 pg/ml in air-exposed, Fig. 6d). This phenomenon was also evident following HDM inhalation (Fig. 6d), although the concentrations of IFN-γ in the HDM-challenged mice were lower than controls (possibly due to the Th2 cytokine production in the allergen-challenged pups). Collectively, these data demonstrate that in utero ETS exposure results in a significant reduction in the number of NK cells in the lungs and commensurate levels of IFN-γ production.

## Discussion

Approximately 300 million people worldwide currently suffer from asthma and its prevalence has increased steadily over the past few decades particularly in children [4]. A large body of epidemiological studies suggest that prenatal and early life environmental exposures, including ETS, have adverse effects on pulmonary function and are important contributors in the development of childhood asthma and allergic disease that can persist into later life [28–32]. Crucially, maternal smoke exposure during pregnancy is associated with wheeze and asthma development after birth even in children not subsequently exposed to ETS [33] implying that the impact of smoke exposure is most prominent in fetal life [34]. However, the mechanisms responsible remain unclear as a consequence of the lack of suitable models to study the adverse effects of in utero ETS exposure. In the present study, we have developed a controlled exposure model to investigate the immunological consequences of prenatal exposure to ETS in order to understand the events responsible for development or exacerbation of allergic airway responses in the offspring. Our data revealed that offspring mice from dams exposed to ETS during gestation displayed negligible peribronchial inflammation and goblet cell hyperplasia in the lungs but exhibited raised serum IgE levels and increased levels of AHR compared to air-exposed controls, even prior to encountering HDM allergen. This shift towards a more allergic phenotype in the offspring exposed to ETS in utero was accompanied with a pronounced exacerbation in the level of eosinophilic inflammation and cell-associated EPO in the airways of both juvenile (7-week old) and adult pups (12 and 18 weeks old) after HDM inhalation. Consistent with these findings, flow cytometric analysis of BALF cells revealed a two-fold increase in the number of CD11b^+^Siglec-F^+^ eosinophils in ETS-exposed offspring when compared to air-exposed pups after allergen inhalation. Interestingly, prenatal ETS exposure alone (i.e. ETS control in the absence of HDM challenge) also caused a small increase in Siglec-F^+^ eosinophils in the airways. Together, these results reveal that prenatal ETS exposure promotes a protracted predisposition to a heightened eosinophilic inflammatory response in the offspring. This protracted pro-allergic phenotype after in utero ETS exposure suggests that the exposure impacted a long-lasting effect on the immune response to allergen in the offspring. Examination of lung tissue by histological analysis revealed a pronounced increase in allergen-induced peribronchial inflammation and mucus production in ETS-exposed offspring when compared to the air-exposed mice. Our study is in agreement with previous findings demonstrating increased AHR and pulmonary inflammation following prenatal exposure [35–39]. Moreover, experimental murine models described by a number of groups reveal that prenatal cigarette smoke exposure favors Th2 differentiation and elevated serum IgE levels [37–40] thus highlighting the important role in utero smoke exposure plays in the development of allergic airway disease. However, the mechanism by which a maternal exposure to ETS predisposes the offspring to allergic inflammation has remained unclear.

To investigate further the immunological consequences of prenatal ETS exposure, we examined the frequency of CD4^+^ T cells and DC in the lungs and the level of airway Th2 cytokines and cysLT in the offspring following allergen inhalation. Monocyte-derived CD11b^+^ DCs have been demonstrated to play an important role in initiating and maintaining allergic Th2 responses to inhaled allergens in asthma [20]. Our results revealed that both CD3^+^CD4^+^ T cells and pulmonary CD11b^+^CD11c^+^MHC-II^bright^ DC were markedly elevated (four-fold) after HDM inhalation in the offspring prenatally exposed to ETS compared to air-exposed progeny. Remarkably, prenatal ETS exposure also caused an exacerbated allergen-induced Th2 cytokine production as well as increased cysLT biosynthesis in the airways. Other reports in both humans and mice revealed that maternal smoke exposure is associated with exacerbated neonatal or postnatal Th2 responses (particularly IL-13 production) [40, 41]. However, despite high levels of IL-4 and IL-13, Singh et al. [40] did not observe an increase in airway mucus production compared to our present study. This discrepancy could be due to differences between the two studies in the allergen or mouse strain used. Evidence implicating Th2 cytokines and IgE synthesis in the pathology of allergic asthma and demonstrating the protective effect of IFN-γ and Th1 cells is well documented [2, 3, 42]. Our data is consistent with prior work in our laboratory demonstrating that in utero ETS exposure of mice alters DNA methylation patterns [43] by causing a significant decrease in IL-13 methylation levels and an increase in IFN-γ [14]. Raised cysLT levels are in agreement with previous studies in man where an increased cysLT production was observed in response to inhaled cigarette smoke [44, 45]. Both Th2 cytokines and cysLT have long been shown to be elevated in asthma patients after allergen challenge [3, 46, 47], and urinary LTE_4_ levels are found to be enhanced during asthma exacerbations [48]. Our data not only demonstrates that prenatal ETS exposure results in a marked increase in CD11b^+^ DC and CD4^+^ T cell numbers in the lungs, but that this response is accompanied by an exacerbated airway Th2 cytokine production and cysLT biosynthesis in the offspring.

Notably, the exposure of pregnant mice to ETS had long lasting effects on the natural immunity of the offspring. A striking observation was that the ETS-induced pro-allergic inflammatory response in the progeny was associated with approximately two-fold reduction in the number of CD3^−^CD19^−^NK1.1^+^CD94^+^ NK cells in the lungs. Moreover, lung NK cells from resting ETS-exposed offspring produced significantly less IFN-γ following NK1.1 cross-linking, a property that typifies fully functional licensed NK cells [49]. The decrease in IFN-γ is likely a consequence of lower NK cell numbers. The reduced number of pulmonary NK cells was a stable defect observable at all ages examined and still evident following the onset of HDM-induced inflammation. Age is an important consideration in NK cell biology because the generation of NK cells in mice is known to be age-dependent with numbers in the spleen reaching detectable levels by 2–3 weeks of age and approximating levels in adults by 6 weeks of age [50]. The coincident reduction of NK cell numbers in both spleen and lung suggest that the maternal ETS exposure impacted the generation or maintenance NK cells systemically in the offspring rather than selectively preventing their recruitment to the lung. In humans, cigarette smoke has been shown to suppress NK cell activation and attenuate NK cytotoxic T cell activity [51]. Moreover, smoking compromises IL-15 production and NK cell response [52]. Such altered NK cell function in smokers is thought to play a role in enhanced susceptibility to respiratory infection [53]. It is thus clear from our studies and others that cigarette smoke exposure has deleterious effects on the innate immune system [12]. Collectively, the data suggests that prenatal ETS exposure results in a reduction in the number of pulmonary NK cells and commensurate levels of IFN-γ in the offspring leading to the enhanced allergen responsiveness. This is consistent with our recent work demonstrating that NK cells play a crucial role in suppressing allergic inflammatory responses [15, 16]. Moreover, NK cell deficiency has been reported to result in viral-induced Th2 responses and subsequent development of allergic inflammation [54].

## Conclusions

In summary, our results demonstrate that in utero ETS exposure predisposes offspring to exacerbated allergic pulmonary eosinophilic inflammation, AHR, airway mucus production and elevated serum IgE levels. Moreover, prenatal ETS exposure caused a pronounced increase in the frequency of both CD11b^+^ DC and CD4^+^ T cells in the lungs and a heightened airway type 2 cytokine and cysLT production in the progeny following allergen inhalation. This pro-allergic phenotype was associated with a marked reduction in pulmonary NK cells and commensurate levels of IFN-γ. These results provide insight into how prenatal ETS exposure influence susceptibility to allergic asthma and suggests that NK cells play a key role in modulating this process.

## Abbreviations

AHR: Airway hyperreactivity
BALF: Bronchoalveolar lavage fluid
CysLT: Cysteinyl leukotrienes
DC: Dendritic cells
EPO: Eosinophil peroxidase
ETS: Environmental tobacco smoke
HDM: House dust mite
IFN-γ: Interferon-gamma
Ig: Immunoglobulin
IL: Interleukin
LMC: Lung mononuclear cells
mAb: Monoclonal antibody
NK cells: Natural killer cells
SEM: Standard error of mean
Th2: T helper 2
